# Genome-Wide Characterization of Light-Regulated Genes in *Neurospora crassa*

**DOI:** 10.1534/g3.114.012617

**Published:** 2014-07-21

**Authors:** Cheng Wu, Fei Yang, Kristina M. Smith, Matthew Peterson, Rigzin Dekhang, Ying Zhang, Jeremy Zucker, Erin L. Bredeweg, Chandrashekara Mallappa, Xiaoying Zhou, Anna Lyubetskaya, Jeffrey P. Townsend, James E. Galagan, Michael Freitag, Jay C. Dunlap, Deborah Bell-Pedersen, Matthew S. Sachs

**Affiliations:** * Department of Biology, Texas A&M University, College Station, Texas 77843-3258; † Department of Biochemistry and Biophysics, Oregon State University, Corvallis, Oregon 97331-7305; ‡ Department of Biomedical Engineering, Boston University, Boston, Massachusetts 02215; § The Eli and Edy L. Broad Institute of Harvard and MIT, Cambridge, Massachusetts 02142; ** Department of Genetics, Geisel School of Medicine, Dartmouth College, Hanover, New Hampshire 03755-3844; †† Bioinformatics Program, Boston University, Boston, Massachusetts 02215; ‡‡ Department of Ecology and Evolutionary Biology, Yale University, New Haven; Connecticut 06520; §§ Department of Biostatistics, Yale School of Public Health, New Haven, Connecticut 06520; *** Department of Microbiology, Boston University, Boston, Massachusetts 02215

**Keywords:** *Neurospora*, RNA-seq, light

## Abstract

The filamentous fungus *Neurospora crassa* responds to light in complex ways. To thoroughly study the transcriptional response of this organism to light, RNA-seq was used to analyze capped and polyadenylated mRNA prepared from mycelium grown for 24 hr in the dark and then exposed to light for 0 (control) 15, 60, 120, and 240 min. More than three-quarters of all defined protein coding genes (79%) were expressed in these cells. The increased sensitivity of RNA-seq compared with previous microarray studies revealed that the RNA levels for 31% of expressed genes were affected two-fold or more by exposure to light. Additionally, a large class of mRNAs, enriched for transcripts specifying products involved in rRNA metabolism, showed decreased expression in response to light, indicating a heretofore undocumented effect of light on this pathway. Based on measured changes in mRNA levels, light generally increases cellular metabolism and at the same time causes significant oxidative stress to the organism. To deal with this stress, protective photopigments are made, antioxidants are produced, and genes involved in ribosome biogenesis are transiently repressed.

The ability to sense and respond to light is critical for the survival of most organisms. In *Neurospora crassa*, one of the best studied model systems for light responses, blue light controls many physiological processes including the synthesis of protective photopigments (carotenoids), asexual and sexual spore formation, the direction of sexual spore release, and entrainment and resetting of the circadian clock (Bahn *et al.* 2007; Ballario and Macino 1997; Chen *et al.* 2010; Corrochano 2007, 2011; Herrera-Estrella and Horwitz 2007; Linden and Macino 1997; Purschwitz *et al.* 2006). Underlying these light-regulated physiological processes is the transcriptional control of gene expression.

*Neurospora* perceives blue light through the photoreceptor and GATA zinc finger transcription factor encoded by *white collar-1* (*wc-1*; NCU02356); the chromophore flavin adenine dinucleotide (FAD) is bound by WC-1 and undergoes a transient covalent addition to the protein on illumination (Ballario *et al.* 1996; Chen and Loros 2009; Froehlich *et al.* 2002; He *et al.* 2002; Linden *et al.* 1997b; Sargent and Briggs 1967). WC-1 interacts with the zinc-finger protein WC-2 (encoded by NCU00902) through its Per-Arnt-Sim (PAS) domain to form a heterodimeric transcription factor, the White Collar Complex (WCC) (Ballario *et al.* 1996; Cheng *et al.* 2002; Crosthwaite *et al.* 1997; Denault *et al.* 2001). On light exposure, the WCC can bind to light-responsive elements (LREs) in the promoters of many light-responsive genes to activate their transcription (Cheng *et al.* 2003; Froehlich *et al.* 2002; He and Liu 2005; Olmedo *et al.* 2010a; Smith *et al.* 2010). In addition to WC-1, the blue light photoreceptor VVD, encoded by *vivid* (*vvd*; NCU03967), which is strongly light-induced under the control of the WCC, plays a key role in photoadaptation by desensitizing the WCC-mediated light response, thereby reducing the transcription of WCC target genes (Chen *et al.* 2010; Gin *et al.* 2013; Heintzen *et al.* 2001; Hunt *et al.* 2010; Malzahn *et al.* 2010; Schwerdtfeger and Linden 2000; Schwerdtfeger and Linden 2003; Shrode *et al.* 2001). The *Neurospora* genome sequence revealed several additional putative photoreceptors, including a cryptochrome (*cry*; NCU00582), two phytochrome homologs (NCU04834 and NCU05790), and an opsin (*nop-1*; NCU10055) (Bieszke *et al.* 1999b; Galagan *et al.* 2003). However, the effects of deletion of these candidate photoreceptors on physiology and light-controlled gene expression are subtle (Chen *et al.* 2009; Froehlich *et al.* 2010; Froehlich *et al.* 2005; Olmedo *et al.* 2010b), consistent with a primary role for the WCC in *Neurospora* light signaling cascades (Chen *et al.* 2009).

To better understand gene regulation in *N. crassa* in response to light, several studies have identified light-controlled genes (Chen *et al.* 2009; Dong *et al.* 2008; Lewis *et al.* 2002; Smith *et al.* 2010). Estimates of the number of light-responsive genes based on microarray analyses have varied widely, ranging from 3% to 14% of the genome (Chen *et al.* 2009; Dong *et al.* 2008; Lewis *et al.* 2002), reflecting the use of different microarray platforms, strains, culture conditions, and statistical cut-offs. Light-induced genes can be approximately classified as early response (with peak mRNA levels between 15 and 45 min of light treatment) and late response (with peak mRNA levels between 45 and 90 min of light treatment), consistent with earlier patterns detected from analyses of individual genes (Linden *et al.* 1997a).

More recently, ChIP-seq was used to identify approximately 400 direct targets of light-activated WCC (Smith *et al.* 2010); genes encoding transcription factors (TFs) were overrepresented. This was consistent with expression studies that uncovered a hierarchical network in which the light-activated WCC directly controls expression of early light-induced genes, including some of the TF genes identified as direct WCC targets (Chen *et al.* 2009). Early light-induced proteins, in turn, control the expression of late light-induced genes. Among the early light-induced TFs, SUB-1 (*submerged protoperithecia-1*; NCU01154) is necessary for light-induction of a large set of the late light-inducible genes (Chen *et al.* 2009), and CSP-1 (*conidial separation-1*; NCU02713) acted at a similar place as *sub-1* in the hierarchy, just below WC-1 (Chen and Loros 2009; Sancar *et al.* 2011).

To better understand the organism’s response to light and to comprehensively describe the light-controlled gene regulatory network, we used RNA-seq to identify *N. crassa* transcripts whose levels are responsive to light. We compared RNA-seq data from dark-grown cells with that from cells exposed to light for between 15 and 240 min and identified both known and novel light-induced genes. We also discovered many transcripts whose levels decreased in response to light. This latter class of transcripts is enriched for genes whose products function in ribosome biogenesis; this response to light has not been described previously.

## Materials and Methods

### Strains

Wild-type strains 74-OR23-IVA (FGSC 2489; *mat A*) and ORS-SL6a (FGSC 4200; *mat a*), and single gene deletion strains (Colot *et al.* 2006) ΔNCU00250 (FGSC12215; *mat a*), ΔNCU01862 (FGSC19012; *mat a*), ΔNCU01870 (FGSC13270; *mat a*), ΔNCU02209 (FGSC16054; *mat a*), ΔNCU02265 (FGSC11554; *mat a*), ΔNCU05770 (FGSC11532; *mat A*), ΔNCU07923 (FGSC19046; *mat A*), and ΔNCU08726 (FGSC11044; *mat a*), were obtained from the Fungal Genetics Stock Center (Kansas City, MO).

### Light treatment

Macroconidia were obtained from flask cultures containing 1× Vogel’s medium with 2% sucrose and 2% agar (Sachs and Yanofsky 1991). Conidia were harvested with water, filtered through cheesecloth, and counted with a hemacytometer. For each time point, 200 ml of Bird’s Medium (Metzenberg 2004) was inoculated to a final concentration of 10^7^ conidia/ml and grown in the dark for 24 hr at 25° with orbital shaking (150 rpm). Some of the cultures were exposed to white light using cool white fluorescent bulbs (1200 lux), and cells were harvested in a darkroom at time 0 (dark), 15, 60, 120, and 240 min after light exposure. The cells were harvested by centrifugation (1000*g* for 1 min) using an IEC clinical centrifuge and washed once with ice-cold sterile water (50 ml). The mycelia pad was cut into small pieces (approximately 100 mg/piece) with a razor. Individual pieces were placed into 15-ml screw-cap tubes and snap-frozen in liquid nitrogen. Mycelia were stored frozen at −80°.

### Total RNA and poly(A) RNA isolation

Total RNA was isolated from frozen mycelia (approximately 100 mg) using 1 g of autoclaved Zirconia/Silica Beads (product number 11079105Z; Biospec Products, Inc, Bartlesville, OK) and a Mini-BeadBeater-8 (Biospec Products, Inc, Bartlesville, OK) with ice-cold 580 µl extraction buffer (100 mM Tris-HCl, pH 7.5; 100 mM LiCl; 20 mM DTT), 420 µL phenol, 420 µL chloroform, and 84 µL 10% SDS (Sachs and Yanofsky 1991). Immediately after removal from −80° storage, mycelia were homogenized in the bead beater for 1 min. After a 4-min end-over-end rotation, the homogenate was centrifuged at 12,000*g* at 4° for 1 min to separate phases. The aqueous phase was extracted with phenol/chloroform and chloroform and precipitated in 0.3 M sodium acetate and ethanol. The pellet was washed twice with 70% ethanol that was prepared with diethylpyrocarbonate (DEPC)-treated water, briefly air-dried, and then dissolved in 100 μl filter-sterilized DEPC-treated water. RNA concentration was determined using a Nanodrop spectrophotometer, and quality was assessed by denaturing gel electrophoresis in formaldehyde gels and northern analyses. Poly(A) mRNA was purified from total RNA using oligo-dT cellulose (Sachs and Yanofsky 1991) for replicate 1, or using the Poly(A)Purist MAG kit (replicate 2) (Ambion, Grand Island, NY). Residual DNA was removed from poly(A) mRNA using Ambion Turbo DNase and intact mRNA was selectively enriched using Epicentre mRNA-Only (Illumina, San Diego, CA) to degrade mRNA species lacking a cap. Poly(A) mRNA concentration was determined by RiboGreen fluorometric assay (Life Technologies, Grand Island, NY).

### cDNA preparation

The procedure for preparing cDNA was modified from a previously described procedure (Carninci *et al.* 2002) as follows. To synthesize the first-strand cDNA, 1 µg poly(A) mRNA was mixed with 1.2 µl of 10 mM dNTPs, 1 µg of random hexamer, heated at 65° for 5 min, and then placed on ice for 3 min to remove any secondary structure. Then, 10 µl of 5× first strand buffer (250 mM Tris-HCl, pH 8.3; 375 mM KCl; 15 mM MgCl_2_), 4.55 µl of 0.1 M DTT, 5.6 µl of 80% glycerol, 2.88 µl of 1 µg/µl BSA, 20 units of RNasin (Promega, Madison WI), and 300 units of SuperScript III reverse-transcriptase (Life Technologies) were added to the reaction tube and the reaction was incubated at 25° for 5 min, 50° for 50 min, and 70° for 15 min in a thermocycler. The reaction mix was precipitated with 0.3 M sodium acetate and ethanol, and the pellet was washed twice with 70% ethanol, briefly air-dried, and dissolved in 22 µl of DEPC-treated water. The RNA–DNA hybrid was treated with 2.5 units of *E. coli* RNaseH (New England Biolabs, Ipswich, MA) at 37° for 20 min. Then, 10 µl of 10× second strand buffer (200 mM Tris-HCl, pH 8.3; 350 mM KCl; 50 mM (NH_4_)_2_SO_4_; 10 mM MgSO_4_; 10 mM MgCl_2_; 0.5% Triton X-100), 3 µl of 10 mM dNTPs, 6 units of RNaseH, and 50 units of *E. coli* DNA polymerase I (New England Biolabs) were added to the reaction tube. The reaction was incubated at 16° for 2.5 hr and then stopped by freezing.

### Sequencing library preparation

cDNA was resuspended in 300 µl TE in a 1.5-ml TPX micro tube (Diagenode, Denville, NJ) and sheared using the low energy cycle for 15 min (15 sec on/15 sec off) in a Diagenode Bioruptor, whose water bath was maintained at 4° with a recirculating chiller. Sheared cDNA was transferred to a 1.7 ml polypropylene microcentrifuge tube and precipitated with 0.3 M sodium acetate and ethanol. The pellet was washed once with 70% ethanol, air-dried, and dissolved in 15 µl sterile water and stored at −20°. The yield of recovered DNA was determined by PicoGreen fluorimetric assay with λ DNA (New England Biolabs) as the standard using a Victor^3^V 1420 multilabel counter (Perkin Elmer, Waltham, MA). An aliquot of sheared DNA (1.5 µl) was examined on a 1% TAE agarose gel to verify sizes ranging between 100 and 800 bp.

RNA-seq libraries were prepared from 1 µg of sheared DNA using an Illumina (San Diego, CA) TruSeq Sample Prep kit according to the manufacturer’s instructions. Each sample was ligated to an indexing adapter and selectively enriched through a 10-cycle amplification. The library was then separated on a 1% TAE agarose gel, and DNA ranging between 200 and 500 bp was excised and purified using QIAEX II gel purification kit (Qiagen, Germantown, MD). The yield of amplified DNA was determined using the PicoGreen assay. An aliquot (3 µl) was examined on a 1% TAE agarose gel to monitor amplification. The DNA was diluted in water to a final concentration of (10 nM in 30 µl) for Illumina sequencing.

### High-throughput sequencing

cDNA libraries were sequenced on an Illumina HiSequation 2000 for 57 cycles and processed with Illumina pipeline RTA 1.13.48 and CASAVA v1.8.2. Trimming the 6-mer adapter generated 51-mer reads. The yield for each sample is shown in Supporting Information, Table S14. Raw and processed data were submitted to the NCBI GEO database with accession number GSE53534.

### Data analysis

Illumina reads were mapped to *N. crassa* assembly 10 with TopHat v2.0.5 (options -i 30 -I 2000). FPKM and fold-changes were calculated using the cuffdiff command from version 2 of the Cufflinks suite. Version 10.6 of the *N. crassa* annotation was used, and fragment bias (the “-b” option) and multiple-read correction (the “-u” option) options were enabled.

Quantitation and statistical tests were performed both separately for each time course as well as for the combined set of replicates. FPKM values across the light time course were hierarchically clustered and visualized as heat maps with Cytoscape 2.8.3 and the clusterMaker plugin (Morris *et al.* 2011). Functional enrichments for each cluster were calculated using the FunCat database (FunCatDb) (Ruepp *et al.* 2004). Gene identifiers in FunCatDb were mapped to the identifiers in the current annotation using the mapping information available at the Broad Institute *Neurospora* Database (http://www.broadinstitute.org/annotation/genome/neurospora/MultiDownloads.html). Gene identifiers in the Gene Ontology were mapped using the GO Term Finder tool (http://go.princeton.edu/cgi-bin/GOTermFinder) with a *Neurospora* GO gene association file (go_for_nc12.tsv downloaded from http://www.broadinstitute.org/annotation/genome/neurospora/Downloads.html).

### Quantitative RT-PCR

For qPCR template, 8 ng of cDNA (0.125 ng for quantification of 25S cDNA) was used as in a reaction containing: 1× Platinum Taq PCR Buffer (200 mM Tris-HCl, pH 8.4, 500 mM KCl) (Life Technologies), 2.5 mM MgCl_2_, 0.2 mM dNTPs, 1× ROX Reference Dye (Life Technologies), 1× SYBR Green I (Life Technologies), 500 nM each primer and 5 U/µl Platinum Taq DNA Polymerase (Life Technologies), or 5 U/µl TaKaRaTaq DNA Polymerase (Clontech, Mountain View, CA) in a 20-μl reaction with filtered sterile DEPC-treated water. Amplification was as follows: 50° for 2 min, 95° for 10 min followed by 40 cycles at 95° for 15 sec, and 60° for 1 min; 25S rRNA was used as an internal control for normalization.

### Phenotype analyses

Phenotyping of *N. crassa* single gene deletion strains, including strains containing deletions of genes whose expression was affected by light, was accomplished in an undergraduate research course at Texas A&M University (BIOL452, Fungal Functional Genomics) taught by some of the authors (M.S.S., D.B.P., Y.Z., and R.D.). Phenotyping was performed as described (Colot *et al.* 2006) with minor modifications. Vogel’s minimal medium (VM)/1.5% sucrose/2% agar was used for vegetative growth and synthetic crossing medium (SCM)/1% sucrose/2% agar for development of female sexual structures (Davis and de Serres 1970). VM was supplemented with 2% yeast extract (YE) when indicated. All strains were analyzed in at least triplicate for each phenotype. All strains were incubated at either 25° or 37° in Percival chambers with a 12-hr light/12-hr dark cycle. Images of hyphae were obtained using Olympus SZX16 microscopes with IDEA 5 cameras and IDEA SPOT software. Plate photos were obtained using Canon APS-C sensor cameras. Tests for female fertility were accomplished using 3 ml SCM in Falcon T12.5 Tissue Flask (353108) with the arrow on the flask’s cap pointed to the top of the flask to ensure sufficient airflow.

## Results

### RNA-seq to identify light-regulated transcripts

**Figure 1:**
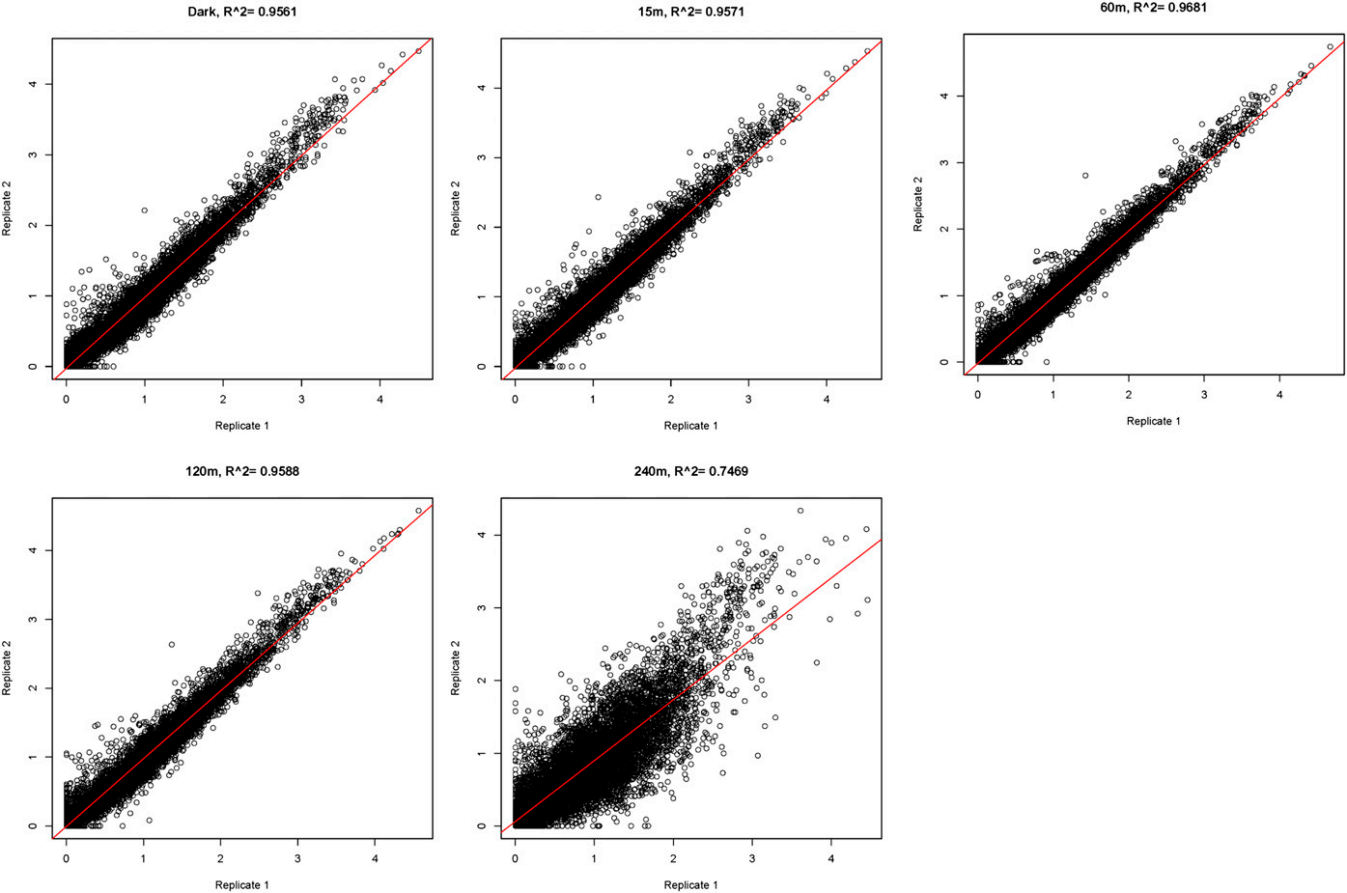
Comparison of RNA-seq replicate experiments. The FPKM for biological replicate 1 is plotted against biological replicate 2 for each gene, demonstrating strong correlation between replicate experiments at each time point. The correlation coefficient, R, is shown for each time point.

We grew *N. crassa* in liquid cultures in the dark for 24 hr and then exposed the cultures to light for 15, 60, 120, or 240 min. Capped and polyadenylated mRNA was purified from harvested cells, hexanucleotide-primed cDNA was produced and sequenced, and results of two independent biological replicates are reported here. The reproducibility between biological replicates was excellent for cultures grown in the dark or exposed to light for 15, 60, and 120 min (R^2^ > 0.95); there was more divergence between the replicate cultures exposed to light for 240 min (R^2^ = 0.75) (Figure 1). For determining expression levels of individual genes, and for statistical analyses, the data from the biological replicates were pooled and analyzed with CuffDiff 2.0 (Trapnell *et al.* 2013). The analyses of the combined data are given in Table S1; the analyses of each independent experiment are given in Table S2 and Table S3. Based on the analyses of the combined data, 79% of predicted protein coding genes (7660 of 9728) were expressed with fragments per kilobase per million reads (FPKM) > 1 at one or more time points analyzed, and these were considered “expressed genes” under these growth conditions. Most expressed genes (55%) specified proteins that have known functions in *N. crassa* or other organisms or whose sequences are conserved in the fungi or other organisms. In contrast, only 16% (341 of 2068) of the poorly or nonexpressed genes (FPKM < 1 at all time points analyzed) specified known or conserved proteins. Importantly, among the poorly expressed genes that specified proteins with known functions were many that had roles in secondary metabolism or were associated with the sexual cycle (Table S4), consistent with their low expression under the vegetative culture conditions used here.

**Figure 2:**
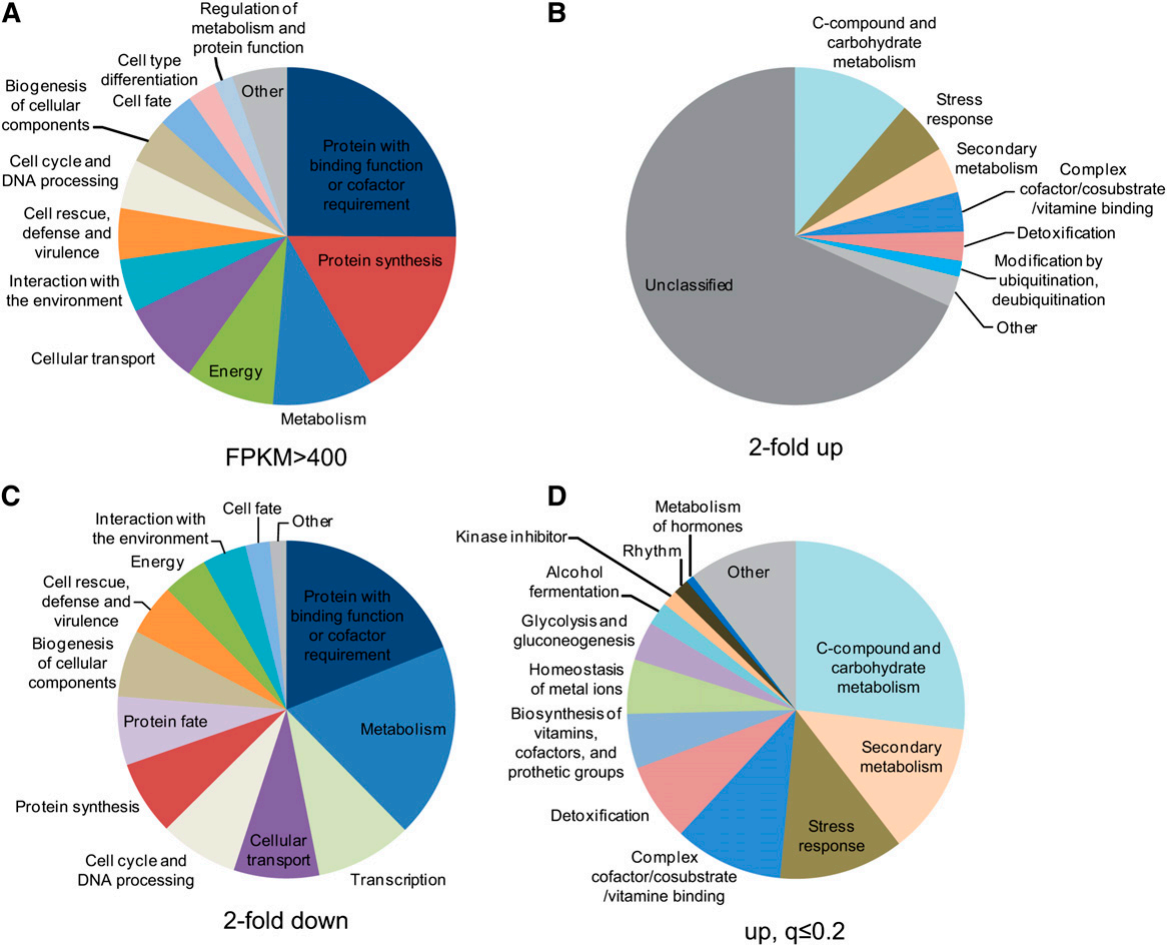
(A) FunCat Analyses of genes with FPKM >400. The complete analyses are given in Table S5. (B, C) FunCat analysis of the most upregulated (B) and most downregulated (C) subsets of the 2353 genes regulated two-fold or more in response to light. The top 999 genes in each category were analyzed. The complete analyses are given in Table S7 and Table S8. (D) FunCat analyses of the upregulated genes whose levels changed with a q-value of 0.2 or less. The complete analyses are given in Table S9.

Our observations of genes abundantly expressed in the dark (and also in the light) were generally consistent with previous work using microarrays (Kasuga *et al.* 2005). The most abundant transcripts in the cell were those implicated in thiamine biosynthesis (NCU06110 and NCU09345) (Cheah *et al.* 2007; Faou and Tropschug 2003; Faou and Tropschug 2004; McColl *et al.* 2003). Also present at high levels in these cultures (FPKM > 400 or FPKM > 1000) were transcripts encoding proteins involved in translation, including those specifying ribosomal proteins and translation factors, and those with roles in energy metabolism (Figure 2A and Table S5).

Light has a major effect on the physiology of *Neurospora* and this is reflected in light-driven changes in the levels of many transcripts. Transcript levels changed at least two-fold for at least one time point with respect to the dark-grown sample in 24% of the predicted protein coding genes (2353 of 9728) or 31% of the expressed genes (2353/7660) (Table S6). Among the transcripts whose levels increased in response to light, the major fraction has unclassified functions in the FunCat scheme (Ruepp *et al.* 2004); genes with roles in carbon metabolism and in stress responses are also among those that are significantly over-represented (Figure 2B and Table S7). Among the transcripts whose levels decreased in response to light, genes with roles in metabolism and biogenenesis were significantly over-represented (Figure 2C and Table S8).

### Cluster analysis of light-regulated transcripts

**Figure 3:**
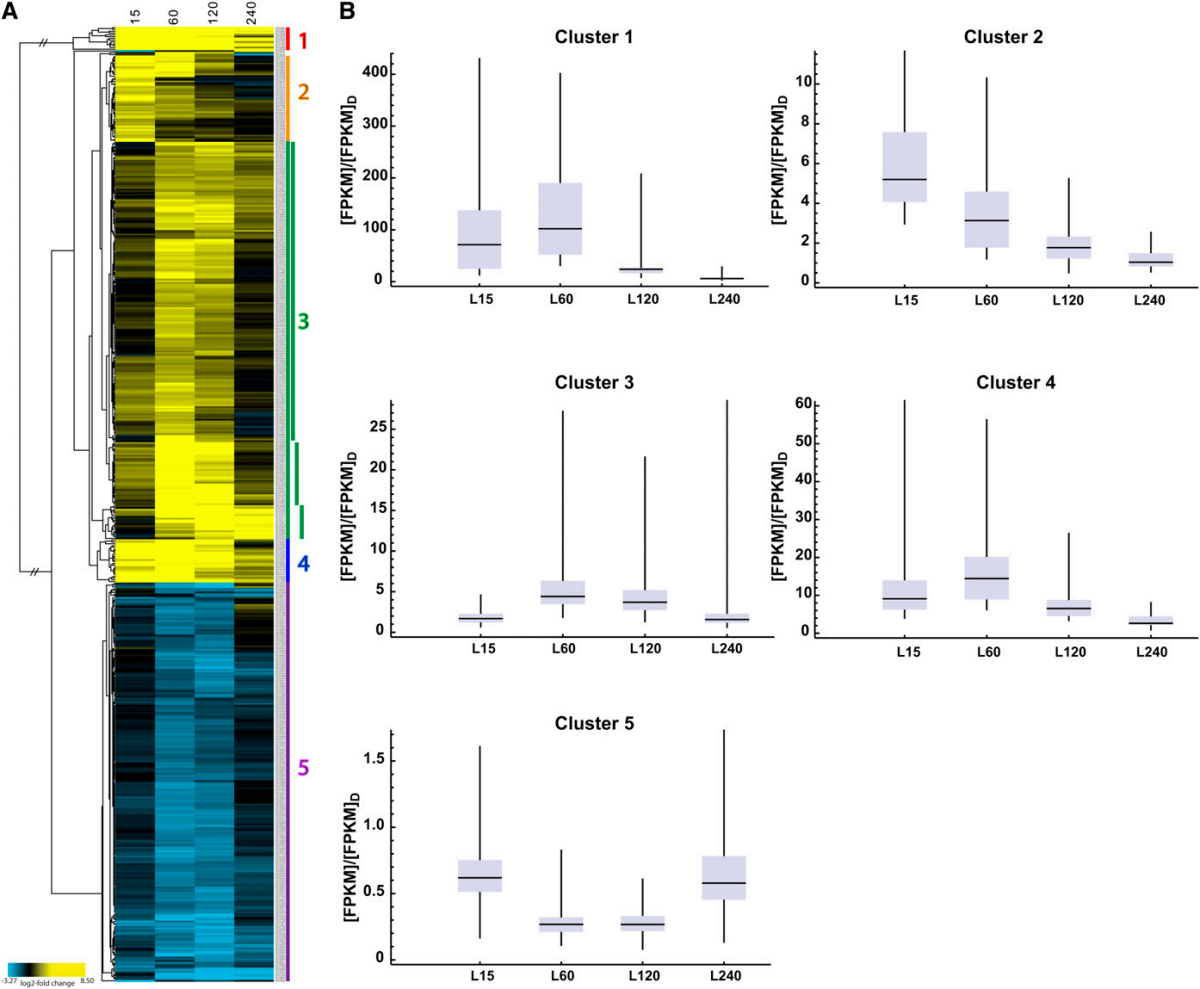

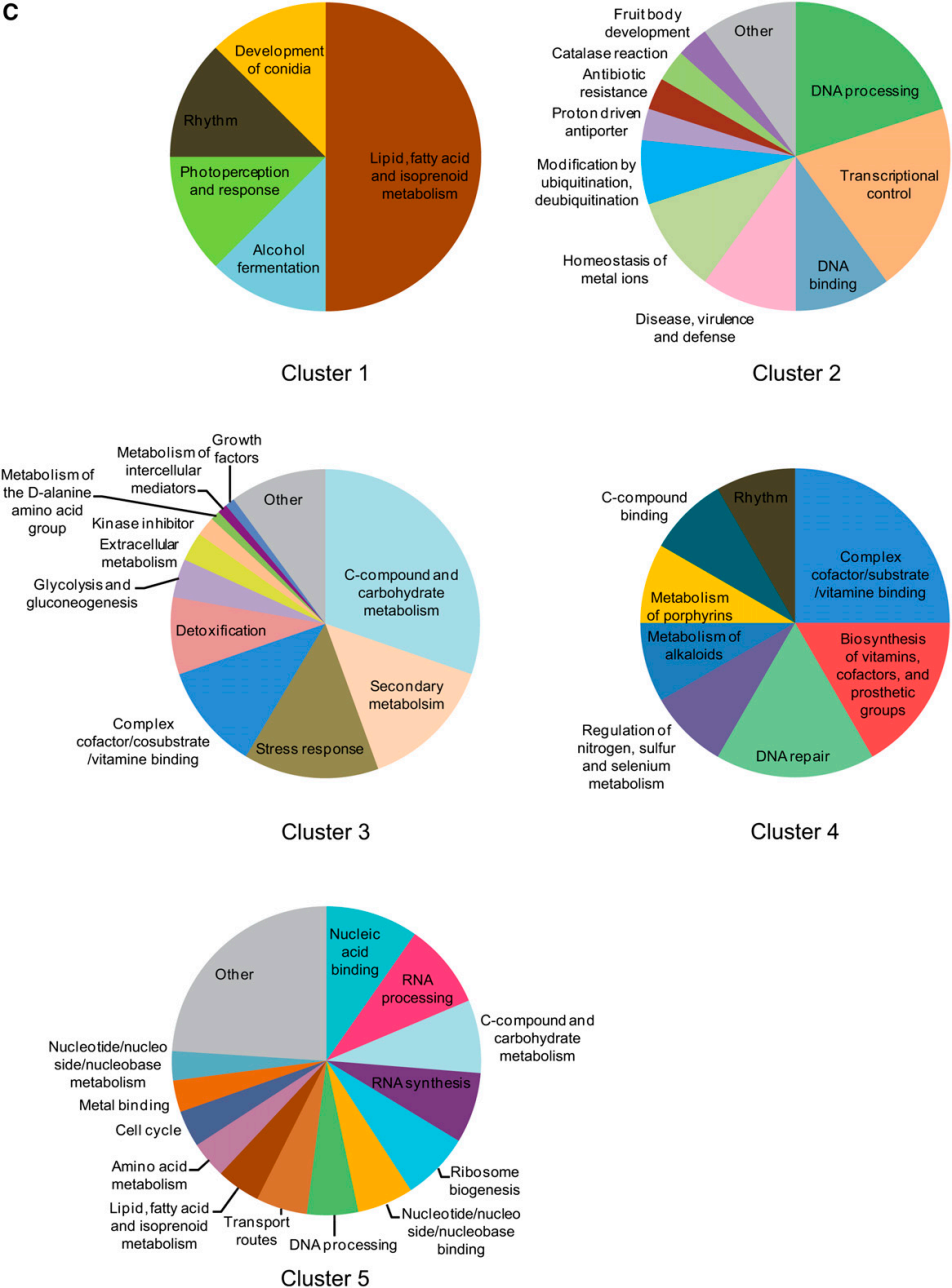
Light responses of selected transcripts. (A) Unsupervised hierarchical clustering of light-inducible responses identifies early and late light-responsive genes for genes whose transcript levels are different with q ≤ 0.2 between cells grown in the dark and exposed to light for 15, 60, 120, or 240 min. Yellow: upregulated; blue: downregulated. Five major clusters determined by visual analysis of the tree structure are indicated; cluster 3 was further divided into three subclusters based on similarities in expression patterns within this relatively large cluster. (B) Expression changes for transcripts in each of the five major clusters. Values for each time point (L15, L60, L120 and L240) are normalized to expression in the dark. The horizontal black bar is the median, the box top and bottom are the 75% and 25% quantiles, and the whiskers extend to the maximum and minimum values. (C) FunCat enrichment analyses of genes in the five major clusters shown in (A). The charts show major enriched classes. The complete data are given in Table S11.

As noted above, a characteristic of the light response is that subsets of genes are regulated with different kinetics, and this suggested that cellular functions might be temporally coordinated. To set the stage for hierarchical clustering of genes by regulation, we identified a subset of transcripts (5%; 532 of 9728 genes) that demonstrated a change in level with a q-value of 0.2 or less in the combined analysis of the two independent experiments (Table S9). The q value provides a measure of the false discovery rate, and 532 genes had q ≤ 0.2, 392 genes had q ≤ 0.1, and 300 genes had q ≤ 0.05 for at least one time point (Table S9). In the aggregate, this subset of 532 genes with q ≤ 0.2 generally reflected the same functional categories as the larger set of 2353 genes with a two-fold change in levels in response to light (compare Figure 2B with Figure 2D and Cluster 5 in Figure 3C), except that unclassified genes are not a major enriched category among the genes whose expression level changes were significant at q ≤ 0.2. Comparisons of genes based on gene ontology (GO) to examine enrichments in the two datasets (all genes that were two-fold regulated by light and all genes whose light regulation met the q ≤ 0.2 stringency requirement) showed overall similar correspondences in GO terms for the top classes (Table S10), with unclassified genes missing in the q ≤ 0.2 set.

Hierarchical clustering based on changes in expression of the 532 gene set relative to the dark sample (Figure 3A) revealed 310 transcripts upregulated and, surprisingly, 222 transcripts downregulated in response to light, the latter representing a class not heretofore described in *N. crassa*. We demarcated genes with common expression profiles into five main clusters based on analyses of overall differences in expression patterns in the tree structure (Figure 3, A and B). The average transcript level of all genes in each cluster at each time point in the light relative to the average transcript level in the dark are given in Figure 3B. FunCat (Ruepp *et al.* 2004) and GO analyses of the genes in each cluster identified significantly enriched functional gene categories (Figure 3C and Table S11).

Cluster 1 includes transcripts with rapid, high-level, sustained light responses. This cluster was significantly enriched for genes involved in carotenoid biosynthesis, such as *albino-1* (*al-1*) (NCU00552) and *al-2* (NCU00585), and blue light photoresponses, including the photoreceptor genes *vvd* and *cry*, both of which attenuate WCC light responses (Heintzen *et al.* 2001; Olmedo *et al.* 2010b). The observation that *vvd* mRNA levels are induced >100-fold whereas the transcripts for its activator WC-1 (Lee *et al.* 2003) are rising and peak later in the light suggests that basal levels of WC-1 are sufficient to maintain photoresponses. The conidiation (con)-related genes *con-10* (NCU07325) and *con-6* (NCU08769) (Berlin and Yanofsky 1985), originally identified by their induction during conidiation and previously shown to be light-responsive (Corrochano *et al.* 1995; Lauter and Russo 1991), are also included in this group.

Cluster 2 represents genes that were typically induced by light within 15 min but returned to dark levels by 60 or 120 min. This response is typical of the light adaptation response of *Neurospora* that is mediated through feedback inhibition of the WCC by the photoreceptor VVD present in cluster 1 (Chen *et al.* 2010; Heintzen *et al.* 2001; Malzahn *et al.* 2010; Schwerdtfeger and Linden 2001, 2003; Shrode *et al.* 2001). Cluster 2 includes WC-1 (NCU02356) and several of the TFs known to be direct targets of WCC: conidial separation-1 (*csp-1*, NCU02713); conidial separation-2, (*csp-2*, NCU06095); *vos-1* (NCU05964); *sub-1* (NCU01154); and siderophore regulation (*sre*, NCU07728) (Chen and Loros 2009; Smith *et al.* 2010). Also included in this group of TFs was the *fluffy* (*fl*) gene (NCU08726) encoding a major regulator of conidiation in *N. crassa* (Bailey and Ebbole 1998; Rerngsamran *et al.* 2005). Although *fl* was not observed in general microarray studies to be light-regulated, its behavior in RNA-seq was similar to that observed in directed studies of *fl* expression (Belden *et al.* 2007; Olmedo *et al.* 2010a). Also included in this cluster were genes involved in metal ion homeostasis (*mig-12*, NCU09830; *sre*, NCU07728; and *cax*, NCU07075), uncovering a previously not discerned need for the fungus to control metal ion homeostasis during exposure to light.

Cluster 3 includes genes that typically peaked in expression between 60 and 120 min in the light, the so-called late light-induced genes. Cluster 3 was visually subdivided into clusters 3a, 3b, and 3c based on differences in expression patterns within the tree structure of this cluster (Figure 3A and Figure S1). Each of the subclusters showed higher levels of RNA at 120 min than at 15 min, as did the main cluster. FunCat analyses showed differences in functional enrichment categories for these subclusters (Table S11). Cluster 3a is highly enriched for genes involved in metabolism and responses to oxidative stress, including the genes for the well-described detoxification enzymes glutathione-S transferase (NCU05706), glutamate decarboxylase (NCU00678), and NADH cytochrome B5 reductase (NCU03112). This cluster also includes a large number of genes encoding hypothetical proteins (75 genes), suggesting that at least some of these genes function in metabolism or cellular stress responses. Cluster 3b is enriched for genes involved in sugar metabolism. This cluster also included *catalase-1* (*cat-1*, NCU08791), which is important in hydrogen peroxide detoxification following light-triggered production of reactive oxygen species (Wang *et al.* 2007a, b). Interestingly, cluster 3b includes *clock-controlled gene-1* (*ccg-1/grg-1*, NCU03753), a gene of unknown function that is regulated by the circadian clock as well as by light, oxidative stress, and glucose starvation (Loros *et al.* 1989; McNally and Free 1988). Both *cat-1* and *ccg-1* were previously shown to be regulated by the TF ATF-1 (also called ASL-1; NCU01346), which functions downstream of the osmosensing MAPK pathway in *N. crassa* (Lamb *et al.* 2012; Yamashita *et al.* 2008). The *atf-1*/*asl*-1 gene displayed a more than two-fold light induction, peaking at 15 min (Table S6); however, it did not make our stringent cut-off of q ≤ 0.2. Cluster 3c is enriched for genes primarily involved in responses to environmental stress, including heat shock and cell wall integrity stress response pathways. These data are consistent with previous studies demonstrating regulation of these pathways by the WCC complex and the clock (Bennett *et al.* 2013; Smith *et al.* 2010).

Cluster 4 genes were rapidly and highly induced by light, and generally stayed induced over the course of light treatment; this pattern of induction resembles that of Cluster 1. This cluster includes the core circadian oscillator gene *frequency* (*frq*, NCU02265), previously shown to be insulated from photoadaptation (Crosthwaite *et al.* 1995). Also included in this cluster are several oxidoreductases (*mig-3*, NCU04452; NCU01861; and NCU08291) important in redox reactions.

**Figure 4:**
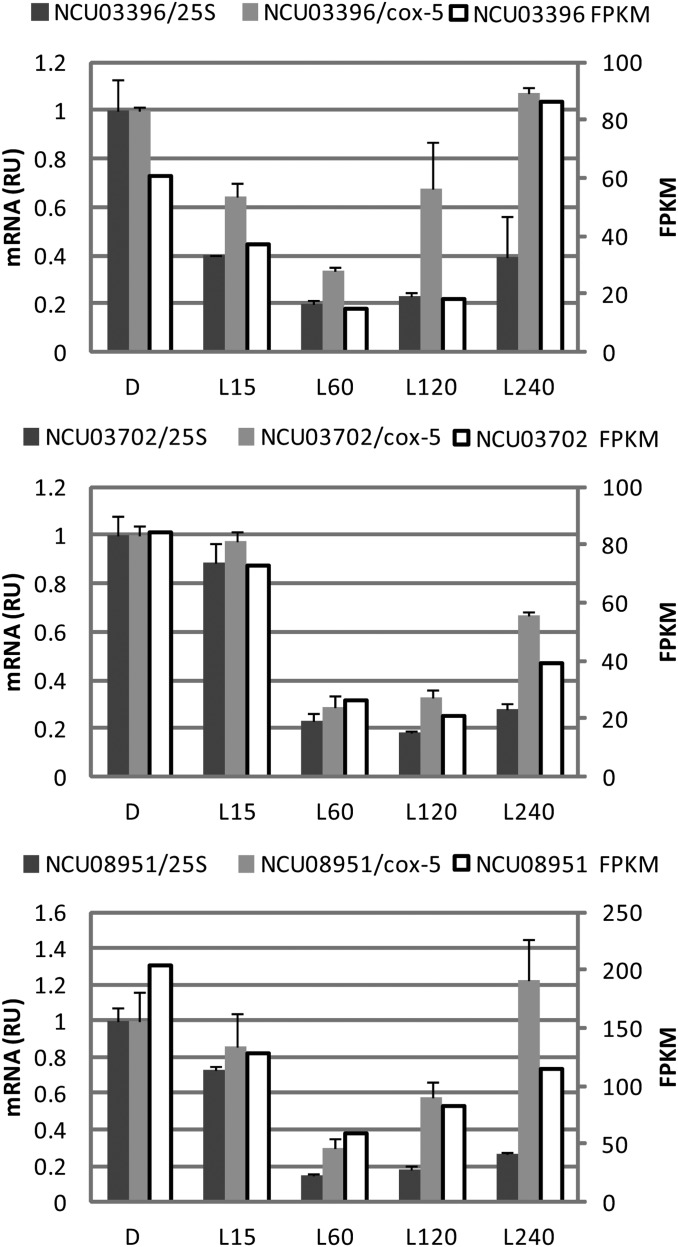
Validation by RT-qPCR of selected downregulated transcripts in Cluster 5. FPKM at each time point (white bars) are compared to RT-qPCR values for each transcript that were normalized to either 25S rRNA (dark gray bars) or to *cox-5* (light gray bars); the *cox-5* transcript does not show a light-response in the RNA-seq data. Error bars show the standard error obtained from triplicate technical replicates of each of the two RNA preparations used for RNA-seq.

Genes in Cluster 5 are repressed by light, with repression being the strongest 60 to 120 min after light treatment. Although some light-repressed genes were noted in previous work (Dong *et al.* 2008), a large cluster of such genes has not been previously described in detail. Because of the novelty of this observation, we confirmed light repression on a subset of transcripts by RT-qPCR (Figure 4). The repression is observed when mRNA levels are normalized either to 25S rRNA levels or to levels of *cox-5*, a transcript whose levels did not respond to light. Genes implicated in ribosome biogenesis were highly enriched in this cluster, suggesting that light triggers a temporary reduction in the production of new ribosomes, which in turn would likely limit protein synthesis. Because light is a stress to the organism (reflected here by the induction of numerous stress response genes in response to light), these data are consistent with previous studies demonstrating global protein synthesis repression following environmental stress (Holcik and Sonenberg 2005; Lindquist 1981; Shalgi *et al.* 2013; Spriggs *et al.* 2010).

Taken together, functional analyses of the clusters revealed that light generally increases cellular metabolism (Clusters 3a and 3b), and at the same time causes significant oxidative stress to the organism. To deal with this stress, protective photopigments are made (Cluster 1), antioxidants are produced (Clusters 2, 3a, 3b, 3c, and 4), genes involved in ribosome biogenesis are transiently repressed (Cluster 5), and the overarching regulatory pattern driven by the circadian system is reset to subjective morning (Cluster 4) in anticipation of a long period of continued light.

### Global regulation by light

**Figure 5:**
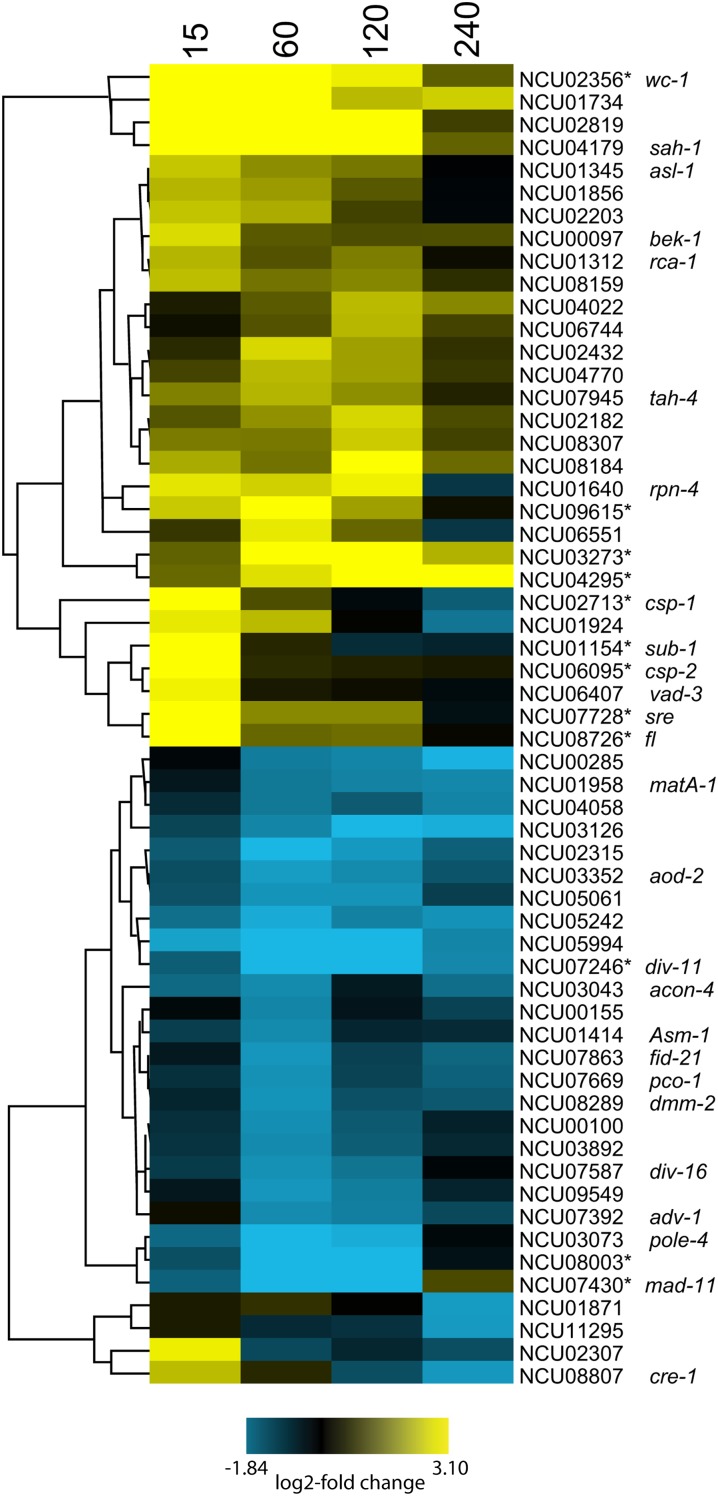
Hierarchical clustering of transcription factors whose mRNA levels are light-regulated. Factors whose mRNA levels were regulated at q ≤ 0.2 are indicated with asterisks.

Nearly 25% of the genome showed a more than two-fold change in gene expression in response to light under our growth conditions. Because previous studies have implicated a hierarchical network of TFs controlling light induction (Chen *et al.* 2009; Smith *et al.* 2010), it was not surprising to find that TFs are over-represented in several of the light-induced clusters. Overall, 58 of 252 identified TFs are regulated by light (Figure 5). Among these, 12 TFs were significantly regulated at a q-value ≤ 0.2 (indicated by asterisks in Figure 5) and are represented in clusters 2, 3a, and 5. Furthermore, of the 27 TFs identified as direct targets of the WCC (Smith *et al.* 2010), 14 showed a more than two-fold change in response to light for at least one time point and 7 with a q-value ≤ 0.2 (Table S12). Of these 14 genes, three TF genes, *adv-1*, NCU07846, and NCU05994, showed decreased levels for at least one time point. Together, these data demonstrate that with the growth conditions used here, not all steady-state mRNAs from genes that are direct targets of the WCC show significant light responses, and that some direct targets of the WCC are repressed by light.

**Figure 6:**
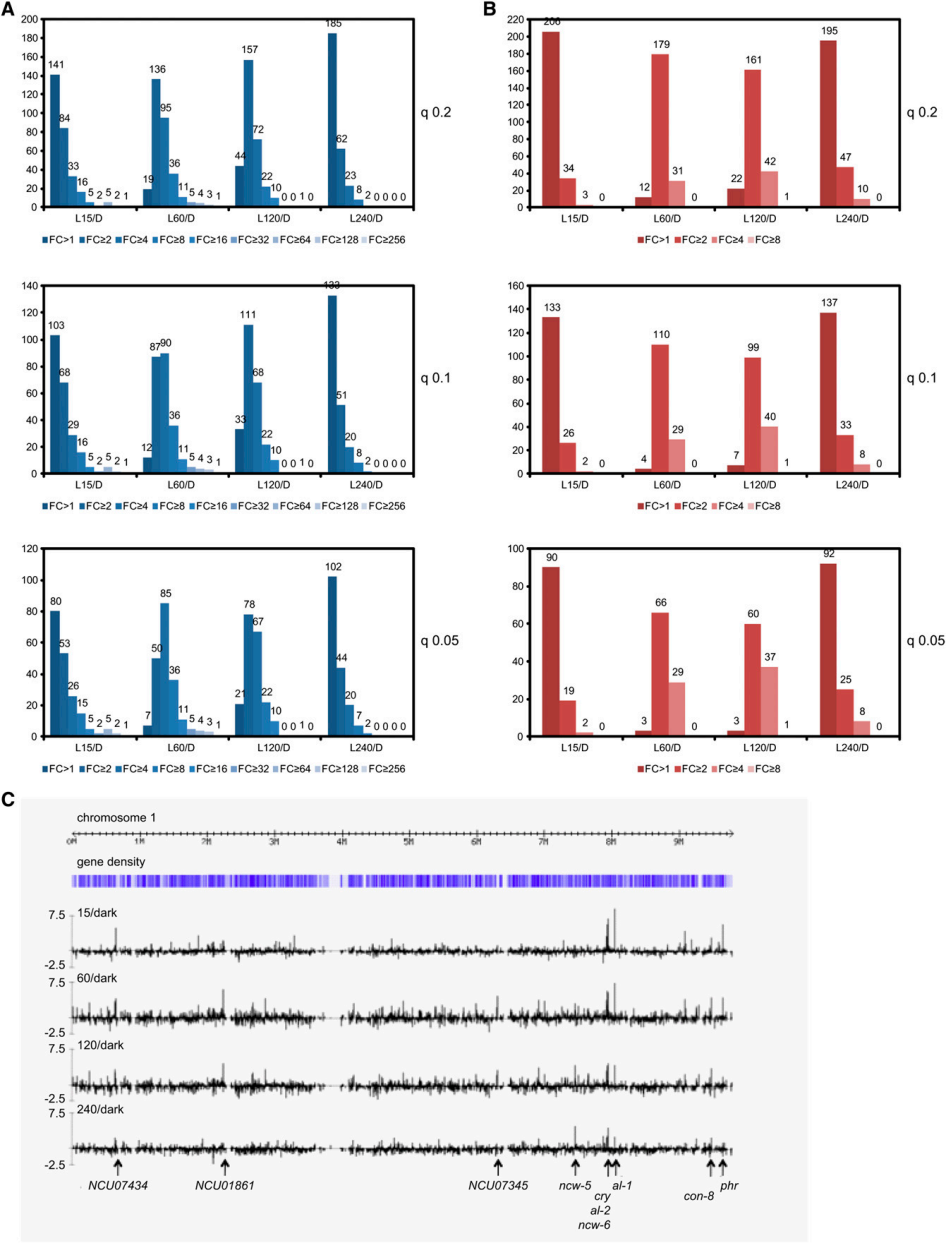
Genome-wide view of light responses. (A) A plot of the number of light-induced genes as a function of time after lights on at each of the time points. These plots show the number of genes for a given level of expression change (FC, fold-change) in response to light (upregulated or downregulated) at q ≤ 0.2, 0.1, or 0.05 (the primary data are in Table S9). (B) A plot of the number of light-repressed genes as a function of time after lights on with all of time points. (C) Pattern of light-regulation of genes on linkage group I (chromosome 1). The log_2_ of the fold-change in expression in the light *vs.* the dark is given on the Y-axis for each time point (15, 60, 120, and 240 min). Several examples of strongly light-induced genes mentioned in the text are marked by arrows.

A relatively large number of transcripts showed sizeable increases in expression on light exposure; 27 mRNAs had 16-fold or greater increases at one or more time points (Table 1). FunCat analysis indicated enrichment for functions similar to those identified for Cluster 1 genes (compare Table S13). Plots of the numbers of genes showing increased or decreased responses to light at different q-value thresholds are shown in Figure 6A. In general, reductions in response to light were smaller than induction in response to light, with no reduction more than eight-fold. The greatest reductions were seen 60 to 120 min after lights on, in contrast to induction, which peaked 15 to 60 min after lights on. Global analyses of the distribution of light-regulated transcripts across *N. crassa* chromosomes did not reveal obvious regions where expression patterns of genes clustered (Figure 6B and Figure S2).

**Table 1:** Transcripts with 16-fold or more induction by light

Locus	Symbol	Name	L15/D	L60/D	L120/D	L240/D
NCU00552	*al-1*	albino-1	8.3	6.9	4.1	2.4
NCU08699	*bli-4*	bli-4 protein	7.5	7.8	4.8	3.5
NCU02190		oxysterol binding protein	7.3	5.6	2.5	1.5
NCU03967	*vvd*	Vivid	6.6	5.1	4.0	3.1
NCU08770		hypothetical protein	6.4	7.6	2.3	0.7
NCU00582	*cry*	cryptochrome DASH	6.3	4.9	4.4	4.1
NCU10063		sugar isomerase	6.2	4.3	2.8	2.1
NCU08769	*con-6*	conidiation-6	6.0	8.5	7.5	2.4
NCU08626	*phr*	photoreactivation-deficient	5.3	4.0	1.9	1.2
NCU00585	*al-2*	albino-2	5.2	5.3	3.9	2.5
NCU07325	*con-10*	conidiation-10	4.8	7.1	4.3	1.5
NCU11424	*cao-2*	carotenoid oxygenase-2	4.8	4.1	2.8	2.1
NCU07434		short-chain dehydrogenase/reductase SDR	4.6	3.8	1.5	1.7
NCU07267	*bli-3*	blue light-induced-3	4.6	6.8	4.6	2.4
NCU11395		S-(hydroxymethyl)glutathione dehydrogenase	4.0	6.1	4.4	2.7
NCU06420		hypothetical protein	3.8	4.1	2.7	1.3
NCU01060		hypothetical protein	3.5	4.9	3.9	2.8
NCU09049		hypothetical protein	3.4	6.1	4.3	1.3
NCU03506		hypothetical protein	3.1	4.3	3.0	1.5
NCU05844		hypothetical protein	2.7	4.5	2.6	−0.5
NCU01861		short chain dehydrogenase/reductase family	2.2	5.6	4.5	1.1
NCU06597		hypothetical protein	2.0	5.4	3.3	0.6
NCU04823		NADP-dependent alcohol dehydrogenase C	1.6	4.5	3.5	1.0
NCU05652		hypothetical protein	1.4	4.6	3.5	0.7
NCU07345		hypothetical protein	1.2	4.3	3.2	0.4
NCU07337		hypothetical protein	1.2	4.2	4.1	2.1
NCU00716	*ncw-5*	non-anchored cell wall protein-5	−0.3	2.8	4.1	4.3

The log_2_ change in expression in the light *vs.* the dark is given for each time point in the light (15, 60, 120, and 240 min).

**Figure 7:**
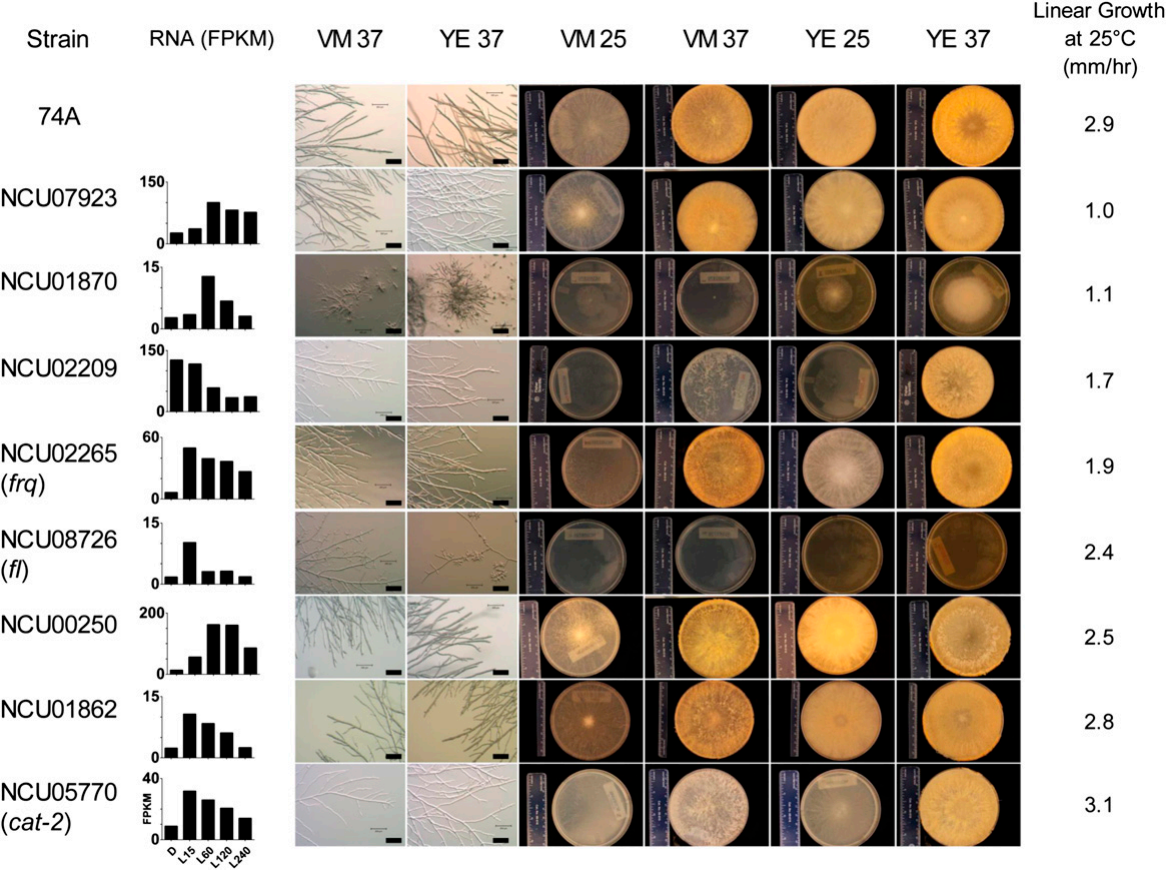
Phenotypes of selected light-regulated genes. A sampling of mutants containing disruptions of genes showing light regulation with q ≤ 0.2 was analyzed for vegetative growth phenotypes. The strain name indicates the gene that was disrupted (74A is the wild-type strain). The indicated gene’s expression in the wild-type strain as determined by RNA-seq is shown for reference. VM: Vogel’s Minimal medium; YE, VM supplemented with yeast extract; 25 and 37, 25°C and 37°C, respectively. Analyses of sexual growth phenotypes showed that each strain was female-fertile, except for NCU01870, which was female-sterile.

We examined the phenotypes of a subset of genes whose transcripts were altered two-fold or more in response to light with a q ≤ 0.2 with both known and unknown functions. The phenotypes of knockouts of these selected genes are shown in Figure 7. Many of these genes showed obvious phenotypes affecting vegetative growth or sexual development. Interestingly, the *frq* deletion strain, in an otherwise WT background, showed slower linear growth in race tubes and a phenotype on plates that differed from the wild-type. This result was surprising given growth differences between *frq*-null mutations in the *ras-1^bd^* background (commonly used for clock studies) and control *ras-1^bd^* strains have not been observed or reported (Aronson *et al.* 1994; Loros and Feldman 1986). We confirmed that an independent disruption of *frq* with the Bar^r^ marker in the wild-type background also showed slower linear growth at 25°; however, in the (already slower-growing) *ras-1^bd^* strain, disruption of *frq* with the Bar^r^ marker did not further reduce the linear growth rate (data not shown). The *fluffy* deletion strain showed a morphological phenotype when yeast extract was included in the growth medium. Although the functions of many of these gene products are hypothetical, the results of phenotyping illustrate that light affects the expression of many genes whose functions are important for the growth of the organism. Additional phenotypic analyses of strains containing deletions of these light-regulated genes should provide clues to the functions of the hypothetical genes and reveal broader connections among the functions of genes with similar expression profiles.

## Discussion

The goal of this work was to achieve a comprehensive understanding of how gene expression in *N. crassa* changes in response to light through the use of RNA-seq. We therefore purified capped and polyadenylated mRNA from vegetatively growing *N. crassa* mycelium grown in the dark or grown in the dark and exposed to light for 15, 60, 120, or 240 min. We sequenced cDNA obtained from this mRNA by using Illumina short-read methodology and analyzed gene expression by measuring the relative abundance of mRNA for known or predicted *N. crassa* protein coding genes (based on assembly Nc10 and annotation 10.6). The abundance of transcripts for approximately 25% of all predicted *N. crassa* genes changed two-fold or more based on these data. Transcript abundance levels for 532 genes (5% of all predicted genes) were light-regulated using a false discovery rate cut-off of q ≤ 0.2 for the data from two independent experiments. The increased power of the RNA-seq approach compared with previous microarray-based approaches enabled the identification of genes that were not highly expressed but that were regulated in response to light. For example, we obtained evidence for a major class of genes predicted to have roles in rRNA processing that were downregulated in response to light.

Estimates of the fraction of *N. crassa* genes induced by light have ranged from 3% to 14% of the predicted protein-coding genes in the genome (Chen *et al.* 2009; Dong *et al.* 2008; Lewis *et al.* 2002). A conservative estimate based on the data obtained here (using a cut-off of q ≤ 0.2) is 5% of the genome, whereas a more liberal estimate is 25% because this is the fraction of genes whose expression changed two-fold in response to light. A major category of genes whose predicted functions are significantly enriched in response to light in the larger group of genes (two-fold regulated) are those with uncategorized or unknown functions. Among these are genes that are fungal-specific. For example, within this category of genes for which there is strong statistical support for light induction is NCU07923 (four-fold induction with q < 0.02) (Table S9), a hypothetical protein that appears strongly conserved within the ascomycetes but not outside of them; deletion of this gene has an obvious vegetative growth phenotype (Figure 7).

While a substantial fraction of predicted *N. crassa* protein-coding genes are regulated at the transcript level in response to light, there were no obvious large chromosomal clusters of genes that showed common regulation. This indicates that the action of light to increase or decrease transcript levels is not generally operating on clustered genes and, further, that these mechanisms are not affecting large contiguous domains of chromatin.

The functions of genes that respond early to light appear different than those that respond later. Light induces the expression of many genes associated with stress responses 60 to 120 min after exposure, and this can be rationalized because light can generate reactive oxygen species (Wang *et al.* 2007b). Consistent with the damaging effects of light, direct targets of the WCC are enriched for DNA repair enzymes (Smith *et al.* 2010). Thus, it is not surprising that we found genes involved in DNA repair mechanisms and encoding light absorbing photopigments are rapidly light-induced. The kinetics of the responses of stress-response genes are similar to those of a subset of genes with roles in C-compound and carbohydrate metabolism whose expression is also induced by light (Cluster 3) (Figure 3C and Table S11). FunCat functional enrichment for this category of genes among late light response genes has been observed previously in microarray studies using the same growth medium (Chen *et al.* 2009). The expression of a large set of genes is also reduced 60 to 120 min after light exposure. Although the mRNAs for the protein components of the ribosome are not reduced by light exposure, many of the mRNAs specifying factors involved in rRNA processing and ribosome assembly are reduced. Thus, it may be more efficient for cells to control ribosome assembly, as opposed to adjusting the levels of abundantly expressed mRNAs encoding ribosome proteins, in response to environmental signals.

Among the early light-induced genes that are associated with DNA repair are NCU08850 (*mus-18*) and NCU08626 (*phr*). Mutations in *mus-18* were originally identified because they were UV-sensitive (Ishii *et al.* 1991), and mutant strains are deficient in excision repair. This light response is deeply conserved because *UVE1*, the homolog of this gene in the basidiomycete *Cryptococcus neoformans*, is also strongly light-induced through the WCC (Verma and Idnurm 2013). UV irradiation can result in the formation of cyclobutane pyrimidine dimers, and *N. crassa phr* specifies a cyclobutane pyrimidine dimer photolyase that reduces this DNA damage through light-dependent photoreactivation (Shimura *et al.* 1999).

The value of RNA-seq in discovery for a better understanding of the behavior of relatively well-characterized genes is illustrated by the results obtained here with *nop-1* (*new eukaryotic opsin 1). nop-1* (NCU10055) encodes a retinal binding protein that affects the expression of genes that are themselves light-regulated, and thus would be anticipated to have light-specific functions (Bieszke *et al.* 1999a, 2007). In previous studies, the *nop-1* mRNA level was not observed to increase early in response to light (Bieszke *et al.* 2007) or early or late in response to light (Chen *et al.* 2009). However, in each of our two independent experiments, and in analyses of the pooled experimental data, *nop-1* mRNA increased at 60 min and 120 min (and came down at 240 in one while remaining up at 240 in the other). This increase in *nop-1* was significant at a q-value <0.2 in each case. The RNA-seq data thus demonstrate that the expression of *nop-1* is light-regulated and provide the basis for further experiments to identify how its increased expression relatively late in the light-response impacts the biology of the organism.

The transcripts for *con-6* (NCU08769) and *con-10* (NCU07325) are strongly induced in response to light as shown here and elsewhere (Chen *et al.* 2009; Corrochano *et al.* 1995; Lauter and Russo 1991; Lauter and Yanofsky 1993; Olmedo *et al.* 2010b). The role of these genes in *N. crassa* has remained elusive because single mutants do not display significant phenotypes. However, a phenotype for the equivalent double mutant of the *A. nidulans* homologs *conF* and *conJ* has been described (Suzuki *et al.* 2013). The double knockout strain resulted in significant increases in the amount of cellular glycerol and erythritol, which delayed conidial germination and provided an increase in resistance of the cells to desiccation. As is the case for *N. crassa*, both *conF* and *conJ* are rapidly light-induced, and the single knockouts displayed no obvious phenotypes. These data suggest the likelihood that *con-6* and *con-10* have redundant functions in spore germination.

Light plays a key role in synchronizing the *N. crassa* circadian clock to local time; therefore, it is not surprising to find that light affects the levels and activities of core clock components. We found that *wc-1* (NCU02356) is transiently light-induced, consistent with previous work (Ballario *et al.* 1996; Linden and Macino 1997). However, although *wc-2* (NCU00902) is reported to be weakly light-induced (Ballario *et al.* 1996; Linden and Macino 1997), no increase in *wc-2* transcripts was observed following light treatment in our experiments. In agreement with these data, light induces a transient increase in WC-1 protein levels, but little or no change in the WC-2 (Schwerdtfeger and Linden 2000; Talora *et al.* 1999), whose levels, unlike WC-1, are not limiting in cells (Cheng *et al.* 2001; Denault *et al.* 2001). Light-activated WCC binds to light-responsive elements (LREs) in the *frq* promoter, leading to subsequent activation of *frq* transcription (Froehlich *et al.* 2002; He and Liu 2005). This change in *frq* mRNA and protein levels is responsible for resetting the phase of the clock to the appropriate time of the day (Crosthwaite *et al.* 1995). Interestingly, none of the other clock components and modifiers of the components, including FRH (FRQ-Interacting RNA Helicase), a binding partner of FRQ necessary for negative feedback (Guo *et al.* 2010), and several kinases (CK1, CK2, PKA, and CAMK-1) and phosphatases (PP1, PP2A, and PP4) that modify the activities of the clock components, met our stringent criteria for light regulation. Three genes, *camk-1* (NCU09123), *ppp-1* (NCU00043 encoding the catalytic subunit of protein phosphatase 1), and *rgb-1* (NCU09377 encoding the regulatory subunit of protein phosphatase 2A), had a more than 2× change in expression following light treatment, but the q-value was >0.2. These data suggest that circadian light responses are mediated by the absolute changes in *frq* mRNA levels through the activity of the WCC.

RNA expression analyses of 27 TF genes shown to be direct targets of the WCC have been accomplished using RT-qPCR to compare RNA levels in dark-grown cells and cells given a 15-min light pulse (Smith *et al.* 2010). These data are similar to the RNA-seq data we obtained (Table S12), with the exception of *adv-1* discussed below. First, in both studies, not all of the genes that are direct targets of the WCC were light-regulated. Second, although not noted before (Smith *et al.* 2010), the steady-state mRNA levels of some of the TF genes are reduced in response to light (*e.g.*, NCU05994). These data suggest that the WCC has repressive as well as activating functions for specific targets, or that other TFs participate in the regulation of the light-repressed genes. Identification of the direct targets of the TFs will help to resolve this question. The gene encoding the ADV-1 TF was the only case in which differences were observed between the two data sets. In our experiments, the levels of *adv-1* mRNA stayed fairly constant in DD and L15 but then decreased more than two-fold at L60 and L120. In contrast, in previous RT-PCR assays, *adv-1* mRNA levels were induced more than two-fold after a 15-min light treatment (Smith *et al.* 2010). The basis for this difference is not understood. It may reflect the use of different media in the two studies or differences in the culture conditions. Specifically, the amount of time the cultures were in the dark before the light treatment varied in the two data sets, 12 hr (Smith *et al.* 2010) *vs.* 24 hr in our experiments, which for a rhythmic gene, such as *adv-1*, would result in time-of-day differences in the dark mRNA levels.

Together, the identification of light-responsive genes will provide the foundation for our ongoing efforts to decipher the specific roles of TFs that respond to light and the clock in the regulation of the global photo-responses and circadian rhythmicity. These studies include determining the direct and indirect targets of the light-controlled TFs, and the interplay of TFs in orchestrating the light and circadian response. With a substantial fraction of the genome showing altered gene expression in response to light, and with different patterns of expression, we anticipate a complex light-controlled and circadian-regulated TF network.

## Supplementary Material

Supporting Information
