# Supplementary material for: Genome-Wide Characterization of Light-Regulated Genes in Neurospora crassa
Source: G3 (Bethesda). 2014 Jul 21;4(9):1731–45. doi: 10.1534/g3.114.012617 (PMC4169166; doi:10.1534/g3.114.012617)
Supplement: Supporting Information [file supp_4_9_1731__index.html]

Genome-Wide Characterization of Light-Regulated Genes in Neurospora crassa — Supporting Information 

# Genome-Wide Characterization of Light-Regulated Genes in *Neurospora crassa*

## Supporting Information for Wu *et al.*, 2014

**Files in this Data Supplement:**

- Supporting Information - Figures S1-S2 and Tables S1-S14 (PDF, 667 KB)
- Figure S1 - Expression-changes for transcripts in each of Cluster 3 subclusters A, B, and C demarcated in (Figure 3). (PDF, 328 KB)
- Figure S2 - Pattern of light-regulation of genes on Linkage Groups II-VII (Chromosomes 2-7). (PDF, 482 KB)
- Table S1 - CuffDiff analyses of FPKM (fragments per kilobase per million reads) for the combined RNA-seq datasets from two biological replicates. The ratio values given are the log2 ratio values. The q-value represents an adjusted p-value that is calculated with a consideration of the false discovery rate of 0.05. (.xlsx, 1 MB)
- Table S2 - CuffDiff analyses of FPKM for biological replicate 1. The ratio values given are the log2 ratio values. (.xlsx, 1 MB)
- Table S3 - CuffDiff analyses of FPKM for biological replicate 2. The ratio values given are the log2 ratio values. (.xlsx, 1 MB)
- Table S4 - Predicted genes that are not expressed in the combined biological replicates (FPKM <1) that are not designated as hypothetical proteins. (.xlsx, 75 KB)
- Table S5 - FunCat analyses for predicted genes that are highly expressed in the dark in the combined biological replicates (FPKM>400 and FPKM>1000). (.xlsx, 62 KB)
- Table S6 - CuffDiff analyses for the subset of transcripts determined to be regulated 2-fold in response to light. (.xlsx, 498 KB)
- Table S7 - FunCat analysis of the 999 mRNAs most up-regulated in response to light. (.xlsx, 65 KB)
- Table S8 - FunCat analysis of the 999 mRNAs most down-regulated in response to light. (.xlsx, 84 KB)
- Table S9 - CuffDiff analyses for the subsets of transcripts determined to respond to light with q≤0.2, 0.1 or 0.05. The ratio values given are the log2 ratio values. (.xlsx, 271 KB)
- Table S10 - Gene Ontology (GO) analyses of genes that were 2-fold regulated by light and genes whose light-regulation met the q≤0.2 stringency requirement. (.xlsx, 39 KB)
- Table S11 - Funcat and GO enrichment analyses of the 5 major clusters delineated in Figure 3A. (.xlsx, 2 MB)
- Table S12 - RNA-seq data (extracted from Table S1) for the 27 TFs analyzed as WCC targets in (SMITH et al. 2010). The ratio values given are the log2 ratio values. (.xlsx, 1 MB)
- Table S13 - FunCat analysis of mRNAs whose levels change more than 16-fold in response to light (Table 1). (.xlsx, 18 KB)
- Table S14 - Summary of read-depth from Illumina RNA-seq. (.xlsx, 9 KB)
