# Supplementary material for: Genome-Wide Characterization of Light-Regulated Genes in Neurospora crassa
Source: G3 (Bethesda). 2014 Jul 21;4(9):1731–45. doi: 10.1534/g3.114.012617 (PMC4169166; doi:10.1534/g3.114.012617)
Supplement: Supporting Information [file supp_g3.114.012617_012617SI.pdf]

G3 · Genes | Genomes | Genetics

# Genome-wide characterization of light-regulated genes in *Neurospora crassa*

Cheng Wu[1], Fei Yang[1], Kristina M. Smith[2], Matthew Peterson[3], Rigzin Dekhang[1], Ying Zhang[1], Jeremy Zucker[4], Erin L. Bredeweg[2], Chandrashekara Mallappa[5], Xiaoying Zhou[5], Anna Lyubetskaya[6], Jeffrey P. Townsend[7,8], James E. Galagan[3,4,6,9], Michael Freitag[2], Jay C. Dunlap[5], Deborah Bell-Pedersen[1], Matthew S. Sachs[1]*

[1]Department of Biology, Texas A&M University, College Station, Texas 77843-3258, [2]Department of Biochemistry and Biophysics, Oregon State University, Corvallis, OR 97331-7305, [3]Department of Biomedical Engineering, Boston University, Boston, MA 02215, [4]The Eli and Edy L. Broad Institute of Harvard and MIT, Cambridge, MA 02142, [5]Department of Genetics, Geisel School of Medicine, Dartmouth College, Hanover, NH 03755-3844, [6]Bioinformatics Program, Boston University, Boston, MA 02215, [7]Department of Ecology and Evolutionary Biology, Yale University, New Haven, CT 06520, [8]Department of Biostatistics, Yale School of Public Health, New Haven, CT 06520, [9]Department of Microbiology, Boston University, Boston, MA 02215

* To whom correspondence should be addressed; E-mail: msachs@bio.tamu.edu

**DOI: 10.1534/g3.114.012617**

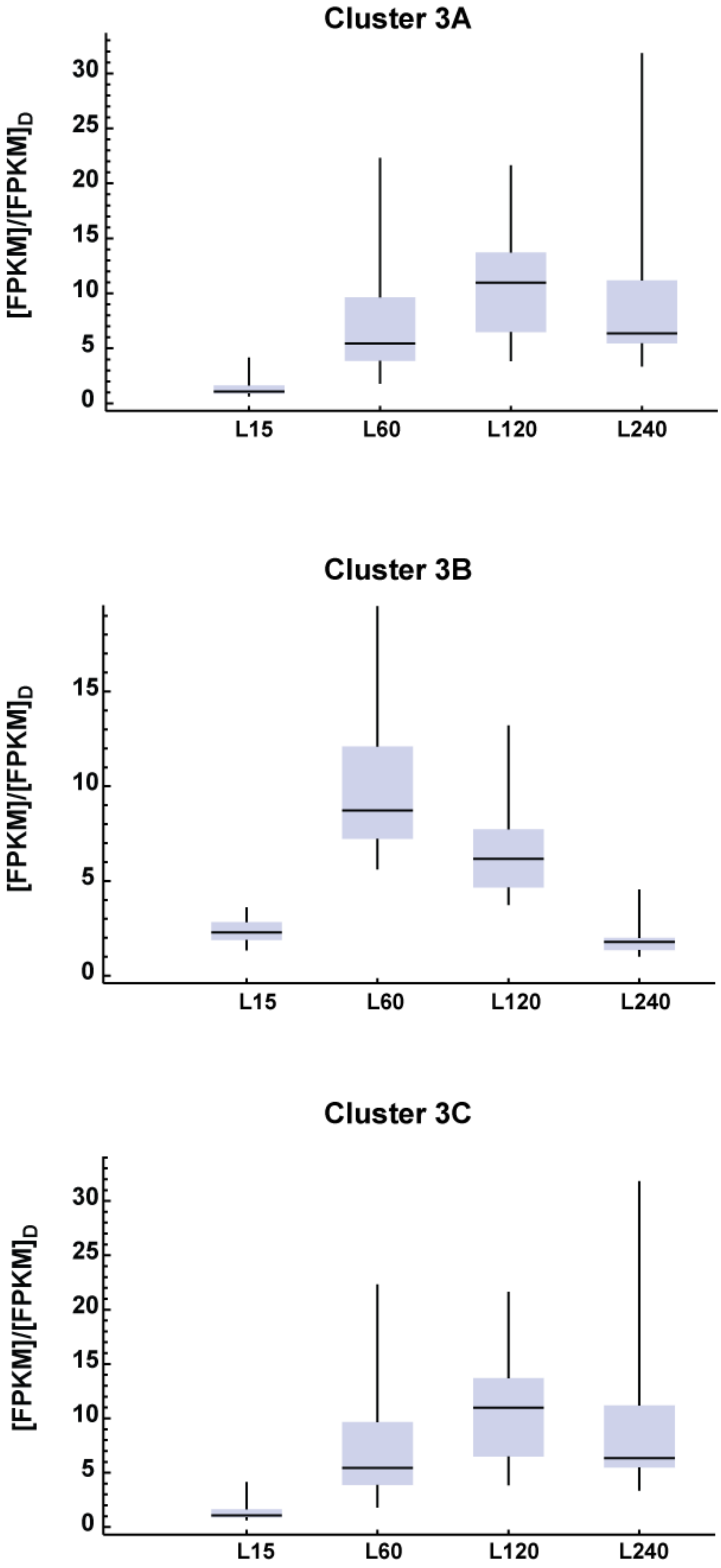


**Figure S1** Expression-changes for transcripts in each of Cluster 3 subclusters A, B, and C demarcated in (Figure 3). Values for each time-point (L15, L60, L120 and L240) are normalized to expression in the dark. The horizontal black bar is the median, the box top and bottom are the 75% and 25% quantiles, and the whiskers extend to the maximum and minimum values.

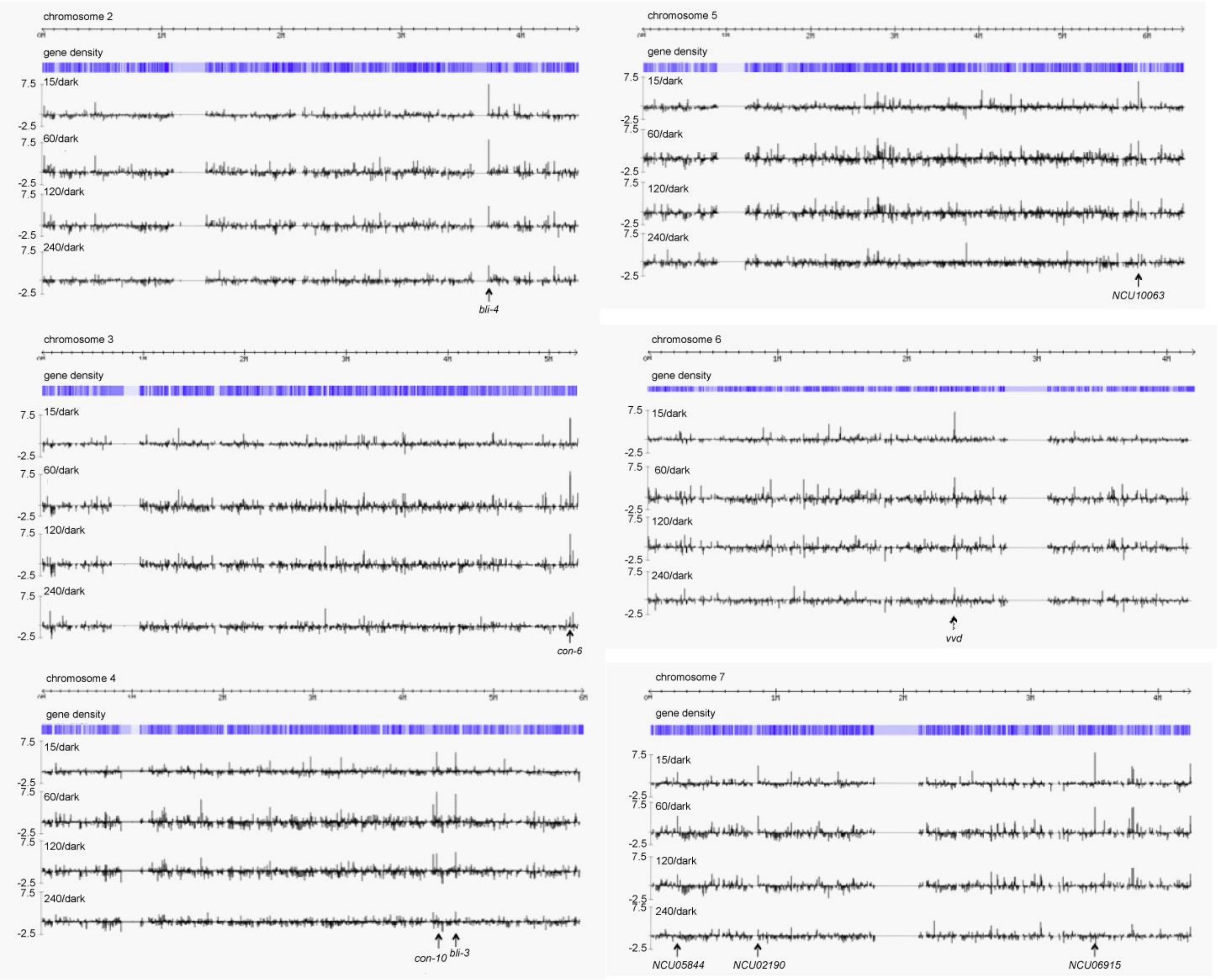
chromosome 2
gene density
15/dark
60/dark
120/dark
240/dark
7.5
-2.5
bli-4
chromosome 3
con-6
chromosome 4
con-10
bli-3
chromosome 5
NCU10063
chromosome 6
vvd
chromosome 7
NCU05844
NCU02190
NCU06915

**Figure S2** Pattern of light-regulation of genes on Linkage Groups II-VII (Chromosomes 2-7). The log-2 change in expression in the light versus the dark is given on the Y-axis for each time-point in the light (15, 60, 120 and 240 min).

**Tables S1-S14**

**Available for download as Excel files at http://www.g3journal.org/lookup/suppl/doi:10.1534/g3.114.012617/-/DC1**

**Table S1** CuffDiff analyses of FPKM (fragments per kilobase per million reads) for the combined RNA-seq datasets from two biological replicates. The ratio values given are the $log_2$ ratio values. The q-value represents an adjusted p-value that is calculated with a consideration of the false discovery rate of 0.05.

**Table S2** CuffDiff analyses of FPKM for biological replicate 1. The ratio values given are the $log_2$ ratio values.

**Table S3** CuffDiff analyses of FPKM for biological replicate 2. The ratio values given are the $log_2$ ratio values.

**Table S4** Predicted genes that are not expressed in the combined biological replicates (FPKM<1) that are not designated as hypothetical proteins.

**Table S5** FunCat analyses for predicted genes that are highly expressed in the dark in the combined biological replicates (FPKM>400 and FPKM>1000).

**Table S6** CuffDiff analyses for the subset of transcripts determined to be regulated 2-fold in response to light.

**Table S7** FunCat analysis of the 999 mRNAs most up-regulated in response to light.

**Table S8** FunCat analysis of the 999 mRNAs most down-regulated in response to light.

**Table S9** CuffDiff analyses for the subsets of transcripts determined to respond to light with $q \leq 0.2$, 0.1 or 0.05. The ratio values given are the $log_2$ ratio values.

**Table S10** Gene Ontology (GO) analyses of genes that were 2-fold regulated by light and genes whose light-regulation met the $q \leq 0.2$ stringency requirement.

**Table S11** Funcat and GO enrichment analyses of the 5 major clusters delineated in Figure 3A.

**Table S12** RNA-seq data (extracted from Table S1) for the 27 TFs analyzed as WCC targets in (SMITH et al. 2010). The ratio values given are the $log_2$ ratio values.

**Table S13** FunCat analysis of mRNAs whose levels change more than 16-fold in response to light (Table 1).

**Table S14** Summary of read-depth from Illumina RNA-seq.
