# Supplementary material for: Genome-Wide Characterization of Light-Regulated Genes in Neurospora crassa
Source: G3 (Bethesda). 2014 Jul 21;4(9):1731–45. doi: 10.1534/g3.114.012617 (PMC4169166; doi:10.1534/g3.114.012617)
Supplement: Supporting Information [file supp_g3.114.012617_FigureS1.pdf]

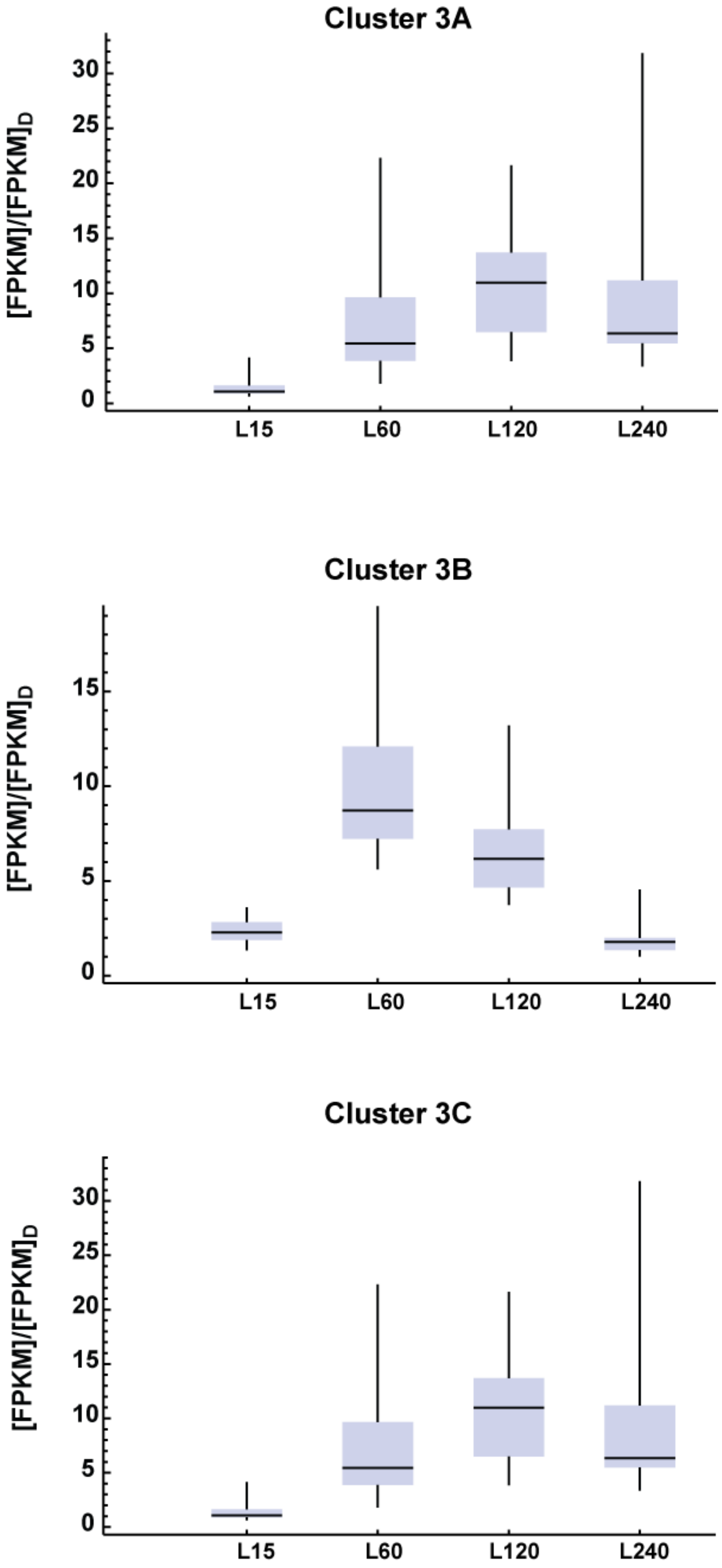

Figure S1 Expression-changes for transcripts in each of Cluster 3 subclusters A, B, and C demarcated in (Figure 3). Values for each time-point (L15, L60, L120 and L240) are normalized to expression in the dark. The horizontal black bar is the median, the box top and bottom are the 75% and 25% quantiles, and the whiskers extend to the maximum and minimum values.
