# Supplementary material for: Genome-Wide Characterization of Light-Regulated Genes in Neurospora crassa
Source: G3 (Bethesda). 2014 Jul 21;4(9):1731–45. doi: 10.1534/g3.114.012617 (PMC4169166; doi:10.1534/g3.114.012617)
Supplement: Supporting Information [file supp_g3.114.012617_FigureS2.pdf]

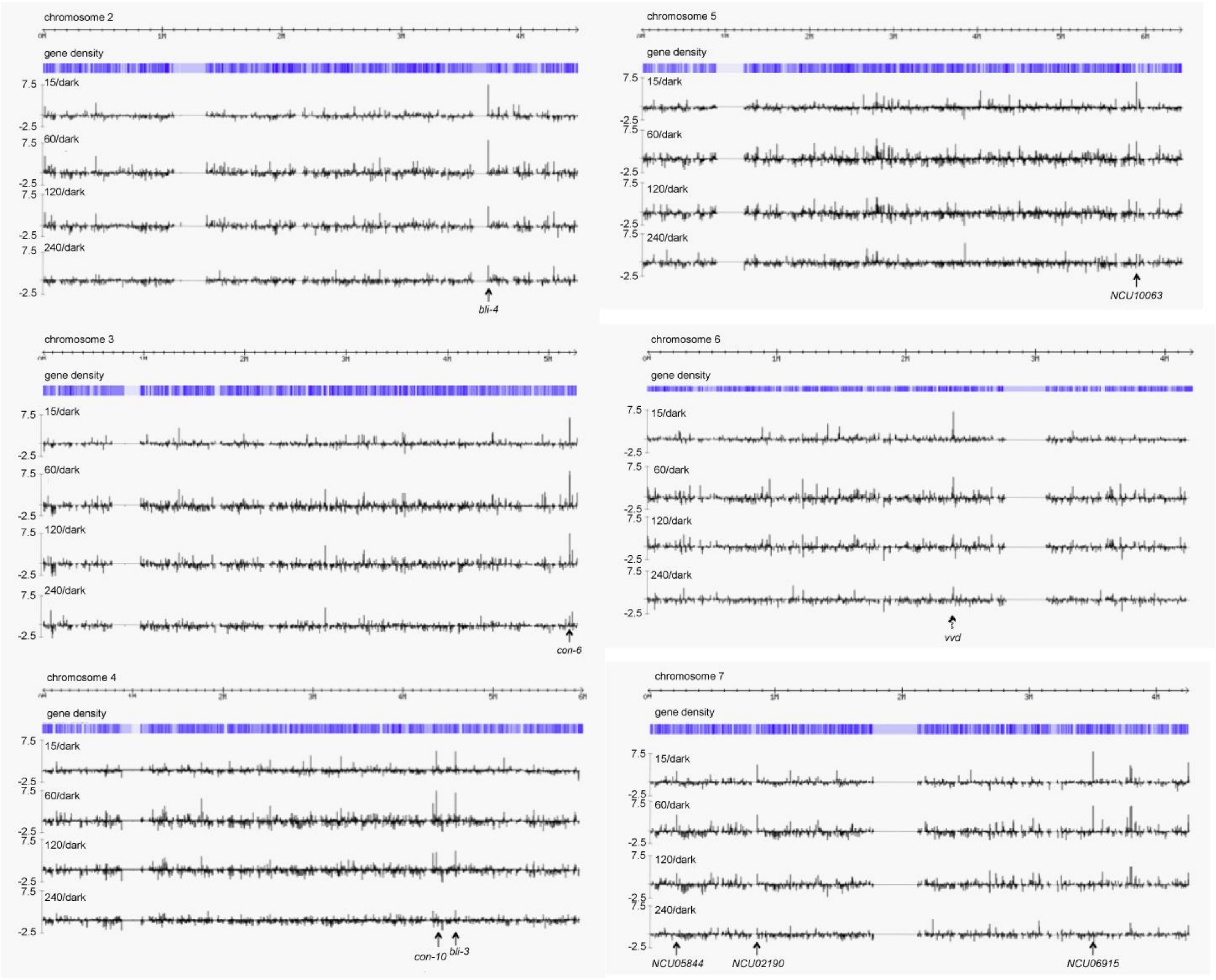
chromosome 2
gene density
15/dark
60/dark
120/dark
240/dark
bli-4
chromosome 3
con-6
chromosome 4
con-10
bli-3
chromosome 5
NCU10063
chromosome 6
vvd
chromosome 7
NCU05844
NCU02190
NCU06915
7.5
-2.5

Figure S2 Pattern of light-regulation of genes on Linkage Groups II-VII (Chromosomes 2-7). The log-2 change in expression in the light versus the dark is given on the Y-axis for each time-point in the light (15, 60, 120 and 240 min).
